# Mutual recognition of qualifications, health workforce migration, and graduate outcomes: a comparative mapping study of undergraduate dental education in Europe

**DOI:** 10.1186/s12960-024-00918-9

**Published:** 2024-06-04

**Authors:** Marie Bryce, Sally Hanks, Lorna Burns, Daniel Zahra, Thomas Gale

**Affiliations:** 1 Peninsula Medical School, University of Plymouth, Plymouth, United Kingdom; 2 Peninsula Dental School, University of Plymouth, Plymouth, United Kingdom

**Keywords:** Dental education, Curricula, Learning outcomes, European Union, Dental workforce migration

## Abstract

**Background:**

The resource needs of health services are served by the recognition of qualifications across borders which allows professionals to migrate between countries. The movement of dentists across the European Union (EU), especially into the United Kingdom (UK), has provided a valuable boost to workforce supply. Recent changes to policy recognising overseas qualifications have brought attention to the equivalence of qualifications awarded in EU countries. Professional regulators need to be confident that dentists who qualified elsewhere have the appropriate knowledge, skills and experience to practise safely and effectively. The aim of this study was to compare UK and EU dental curricula, identify any differences, and compare the extent of pre-qualification clinical experience.

**Methods:**

This was a mixed methods study comprising a questionnaire and website searches to identify information about curricula, competences, and quality assurance arrangements in each country. The questionnaire was sent to organisations responsible for regulating dental education or dental practice in EU member states. This was supplemented with information obtained from website searches of stakeholder organisations for each country including regulators, professional associations, ministries, and providers of dental education. A map of dental training across the EU was created.

**Results:**

National learning outcomes for dental education were identified for seven countries. No national outcomes were identified 13 countries; therefore, learning outcomes were mapped at institution level only. No information about learning outcomes was available for six countries. In one country, there is no basic dental training. Clinical skills and communication were generally well represented. Management and leadership were less represented. Only eight countries referenced a need for graduates to be aware of their own limitations. In most countries, quality assurance of dental education is not undertaken by dental organisations, but by national quality assurance agencies for higher education. In many cases, it was not possible to ascertain the extent of graduates’ direct clinical experience with patients.

**Conclusions:**

The findings demonstrate considerable variation in learning outcomes for dental education between countries and institutions in Europe. This presents a challenge to decision-makers responsible for national recognition and accreditation of diverse qualifications across Europe to maintain a safe, capable, international workforce; but one that this comparison of programmes helps to address.

## Background

The United Kingdom’s (UK) departure from the European Union (known as *Brexit*) carries potentially significant implications for the migration of healthcare workers between EU member states and the UK, not least for professional regulation processes and the accreditation of health workers’ qualifications. Since 2005, as a result of Directive 2005/36/EC [1], healthcare workers in several professional groups, including doctors, dentists, nurses, midwives and pharmacists, who are EU nationals and achieved their professional qualification in the EU, have been free to move across boundaries to work in any EU member state. The Directive sets out that professional qualifications for these healthcare workers must be reciprocally recognised by member states [2]. As a result of Brexit, the terms of the Directive no longer apply to the United Kingdom, and replacement arrangements for the recognition of health professionals’ qualifications are now in place, with a Professional Qualifications Act enacted by the UK parliament in 2022 [3]. This Act gives professional regulators powers to determine whether practitioners seeking to migrate into the UK have ‘substantially the same knowledge and skills, to substantially the same standard’ as those with UK qualifications. This applies at either individual level or through bilateral regulator recognition agreements allowing for mutual recognition of professional qualifications between countries [3].

Migration of dentists from Europe into the UK has been an important contributor to the UK dental workforce in the recent years. At the end of 2021 there were a total of 43 292 dentists registered to practise in the UK, of whom 16% (7091) had qualified in the European Economic Area (EEA), which includes the European Union member states plus Iceland, Liechtenstein and Norway [4]. These numbers have remained largely stable since 2019, when there were a total of 42 470 UK registered dentists among which 16.2% (6881) were EEA qualified [5]. The proportion of EEA new registrants also stayed stable during this time. In 2021 1500 new dentists joined the UK register, of which 29.5% (446) had qualified in EEA countries [4], compared to 22.9% (398 of 1737) in 2019 [5]. This suggests limited impact to date from Brexit or the Covid-19 pandemic. However, prior to this Brexit period, the number of EEA qualified dentists registered in the UK had seen rapid growth, increasing by 214% between 2000 and 2019, compared to an 18% increase in the number of UK graduates on the register [6].

While migrating workers make a significant contribution to UK healthcare provision, migration also presents policy challenges for health professions regulation, especially in relation to the recognition and accreditation of qualifications. Namely, national bodies responsible for recognition and accreditation need to have confidence that migrating professionals’ qualifications are of an equivalent standard to those offered within the country where the migrant health worker will practise. In the UK, dental professionals, including dentists and other dental care professionals such as dental nurses, hygienists, and dental therapists, are subject to statutory regulation by the General Dental Council (GDC). An independent organisation with statutory responsibilities set out in the *Dentists Act 1984* (as amended), the GDC has a number of core functions which it pursues in order to fulfil its overarching objective of protecting the health, wellbeing and safety of the public [7]. Among its responsibilities are the registration of dental professionals meeting its standards, and setting standards for UK providers of education and training. In *Preparing for practice: dental team learning outcomes for registration* [8], the GDC sets out learning outcomes for all the dental professional groups it regulates. For graduating dentists, the document includes six overarching outcomes, plus a further 151 detailed outcomes, divided into four main domains: clinical, communication, professionalism, and management and leadership. Outcomes in each of these four domains are organised around a number of criteria. These learning outcomes constitute the expectations for dental education in the UK, and the GDC operates quality assurance processes of all UK dental training programmes to ensure dental education providers, and therefore the graduates they produce, meet these expectations.

While this quality assurance process assures the standard of UK graduates, a major question for professional regulators such as the GDC, is how to assess the merits of immigrating healthcare workers’ qualifications, and how to establish the extent to which their studies will have equipped them with the training and experience comparable to that of locally qualified graduates. This issue touches on several core functions of professional regulation, including the development of educational standards and accreditation of qualifications; ensuring registration and the right to practise are granted only to those eligible; and ensuring that those registered are safe to practise. For UK regulators, Brexit and the resultant uncertainty over whether current health worker mobility arrangements would be retained, modified or abruptly rescinded, brought questions about the accreditation of qualifications awarded in EU countries to the fore. Developing accreditation processes for this new era will require policymakers to balance regulatory objectives of assuring patient safety, with individuals’ aspirations for mobility, and health service human resource needs. Although Brexit made this a pressing issue for the UK and EU, the question of recognition of qualifications across borders is a perennial concern for health policymakers worldwide.

Set against this policy context, we undertook research to compare UK and EU member states’ dental curricula, to identify where any differences may exist, and to identify the extent of graduate dentists’ pre-qualification clinical experience with patients. Our research also sought to identify what quality assurance processes are in place for dental education in Europe. This paper reports findings from this curricula mapping exercise, and sets out the implications for mutual recognition of health professional qualifications.

## Methods

This study, part of a wider project [9], used a mixed methods approach to mapping basic dental training across the UK and the 27 EU member states, using website searching and a questionnaire to identify key information about curricula, competences, and quality assurance arrangements in each country.

### Website searches

Key stakeholder organisations in basic dental training were identified in each country including independent regulatory authorities, professional associations, Ministries of health or education, and providers of basic dental training. The organisations were identified through a number of online sources such as the EU Manual of Dental Practice [10], the Federation of European Dental Competent Authorities and Regulators (FEDCAR) list of members [11], and the EEA list of competent authorities [12]. Websites for identified organisations were then searched by five researchers (LB, MB, TG, SH, GL) for information relevant to our research questions. A data extraction form was used to ensure consistent approach to searches, and to collate and organise the information retrieved. A hierarchical approach to the searches was followed, so that regulator and competent authority websites were reviewed first, followed by health ministry websites, and finally individual dental education provider websites until information to address all questions was identified.

For each country, the following information on curricula and learning outcomes was sought:

- National professional competences
- Domains of curricula
- Published standards of dental education
- Quality assurance processes

Where information was provided in the language of the host country, online translation or within team language proficiencies enabled the extraction of relevant information. All URLs were recorded and useful documents were saved.

### Questionnaire

We developed a short questionnaire to collect factual information, and elicit additional relevant curriculum documents, about basic dental training and its quality assurance in EU member states. In particular, we sought information about national level curricula or learning outcomes not publicly available online. Between December 2019 and January 2020, the questionnaire was distributed by email to organisations responsible for regulating dental education or dental practice in EU member states, identified from our website searches and the FEDCAR website [11], and also distributed by email via FEDCAR and the Association for Dental Education in Europe (ADEE), to organisations including regulators and dental schools. We received 12 responses to the questionnaire, from organisations in 10 EU member states. Responses were received from national regulating organisations in Belgium, Denmark, Finland, France, Ireland, Spain and Sweden. Responses were also received from dental schools in Luxembourg, Finland, Portugal, Slovenia, and Spain. The responses were used for triangulating the information found via the website searches [9].

### Synthesis and mapping

Information that related to professional competences, domains of curricula, or standards of dental education were mapped against the GDC *Preparing for Practice* framework [13]. Where national level graduate outcomes or a national curriculum for dental education was identified, these were mapped against the domains and criteria of *Preparing for Practice* [13]*.* Where no national outcomes were available, we mapped the curriculum or outcomes of a single dental school as an illustrative example of provision in a country, though with the caveat that this may not be representative of the country’s provision as a whole.

The six overarching outcomes in *Preparing for Practice* plus its four domains (clinical, professionalism, management and leadership, and communication) and their criteria were extracted into a spreadsheet. Given its UK-specific meaning, an additional domain focused on graduates’ recognition of their role as GDC registrants was not included in the mapping exercise. Two senior clinical educators (SH&TG) with expert knowledge of dentistry and learning outcomes reviewed available information on curricula or learning outcomes from each EU member state and cross-referenced it against the *Preparing for Practice* domains and sections.

## Results

**Fig. 1:**
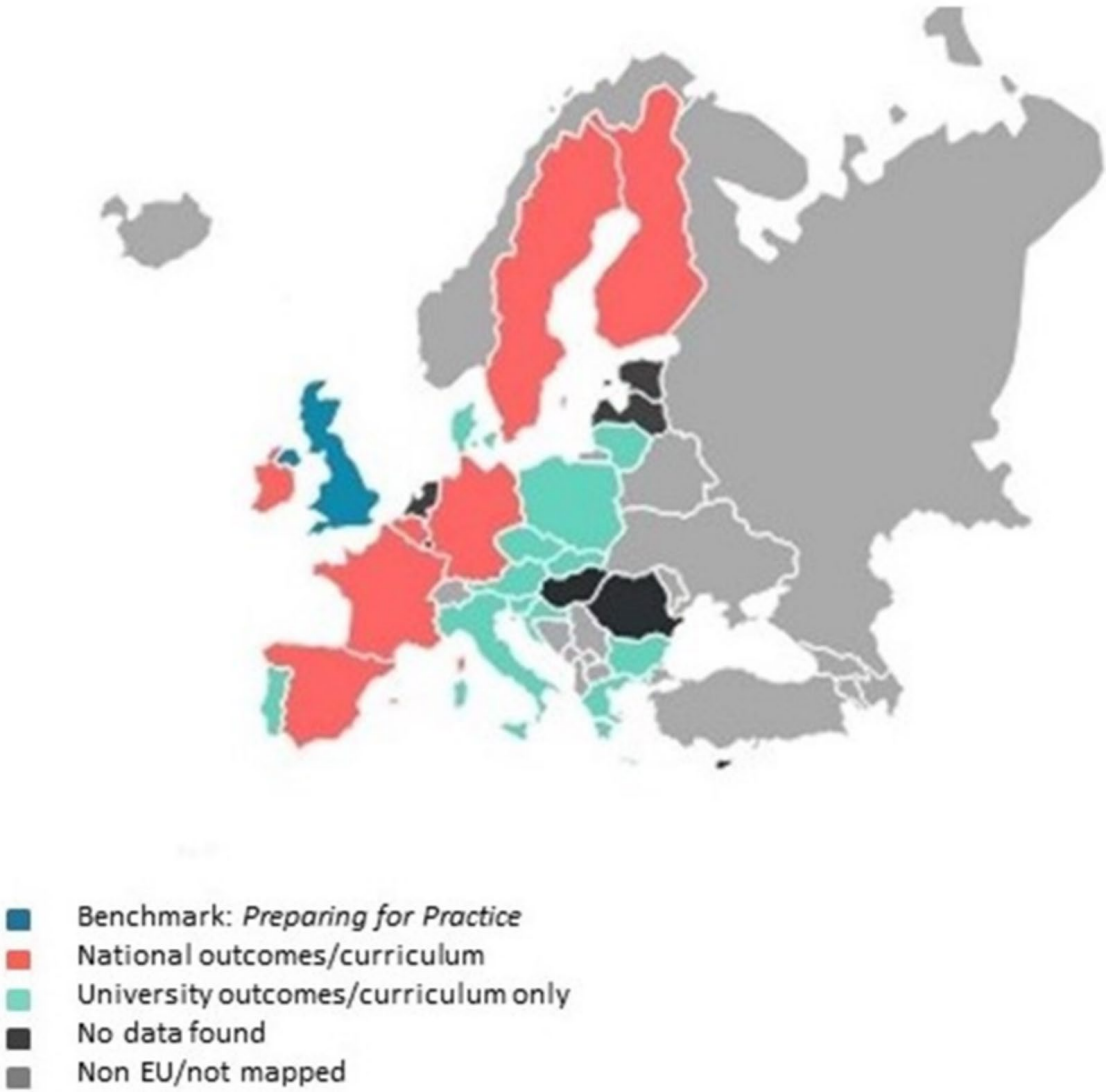
EU member states mapped to preparing for practice outcomes

National learning outcomes for dental education were identified in seven EU member states: Belgium, Finland, France, Germany, Ireland, Spain, Sweden. Finland and Ireland have adopted the competences set out in the Association for Dental Education in Europe (ADEE) document *Profile and Competences for Graduating European Dentist* [14] as their nationally agreed set of professional competences. For a further 13 countries we were able to identify learning outcomes from a single dental school to use as an exemplar. No information about learning outcomes was available for Cyprus, Estonia, Netherlands, Hungary, Latvia, and Romania, although the single Cypriot dental school at the European University of Cyprus stated that its programme uses the ADEE *Profile and Competences for Graduating European Dentist* [14]. Luxembourg has no dental schools. Figure 1 shows EU member states categorised by the level of learning outcomes identified.

Learning outcomes for Germany, Spain and Sweden were mapped at both national and institution level, as country level data was identified through the survey in addition to institution level data for those countries. We retained the institutional level data as it serves to illustrate variation between national and institutional learning outcomes. As shown in Tables 1, 2, 3, the outcomes for dental education set at national level for these countries were not consistently replicated in the curricula of the individual institutions mapped as part of this research.

**Table 1:**
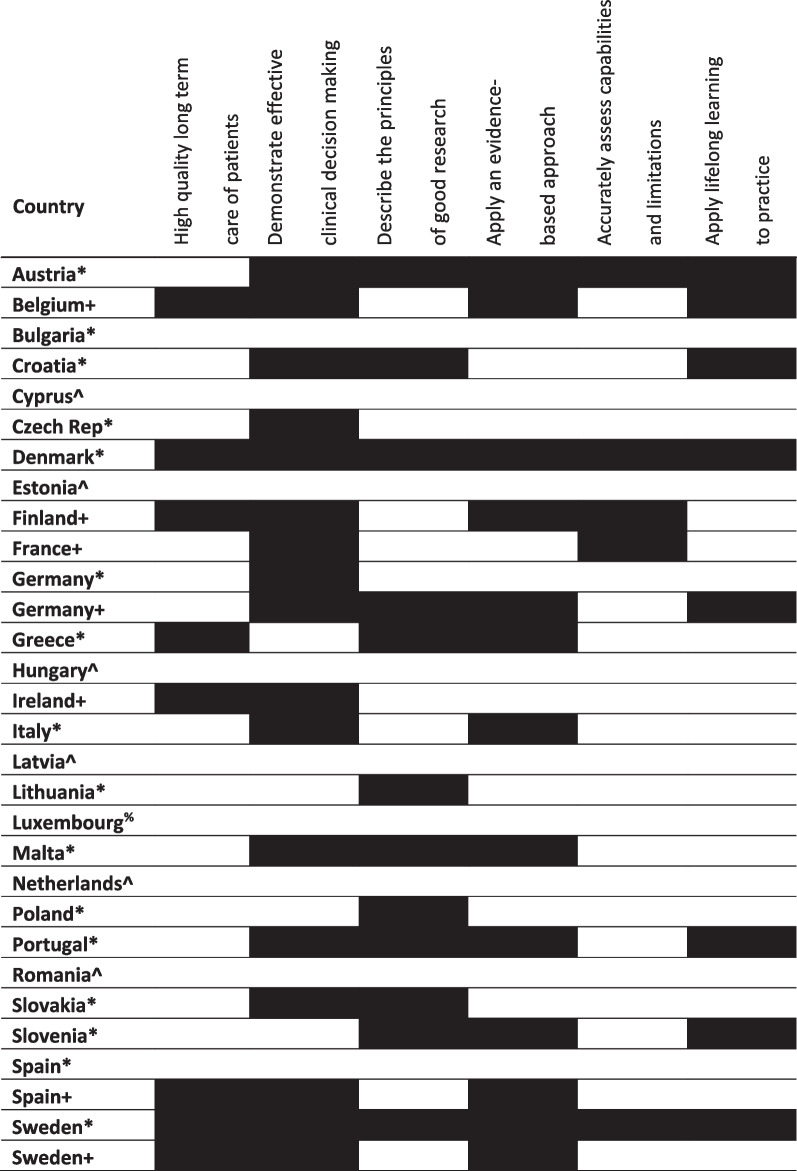
Mapping against overarching outcomes in preparing for practice

^+^Mapped to outcomes or curriculum from a national authority

*Mapped to outcomes or curriculum from a single dental school/university

^%^No dental school

^^^No information identified

**Table 2:**
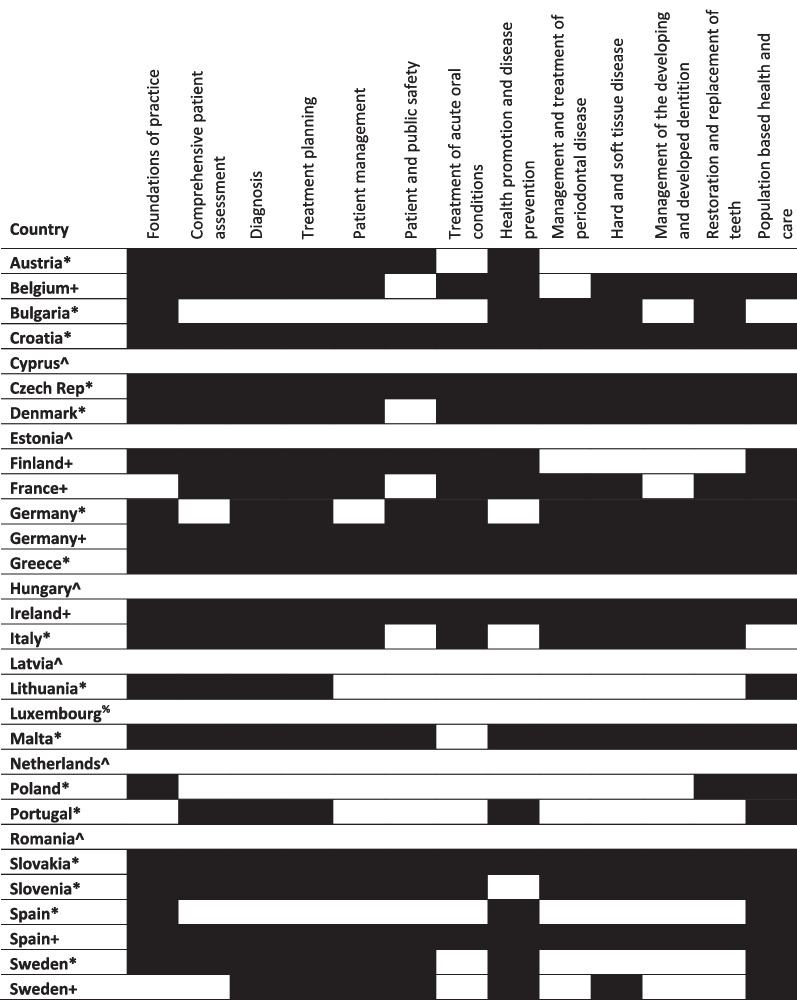
Mapping against clinical outcomes in preparing for practice

^+^Mapped to outcomes or curriculum from a national authority

*Mapped to outcomes or curriculum from a single dental school/university

^%^No dental school

^^^No information identified

**Table 3:**
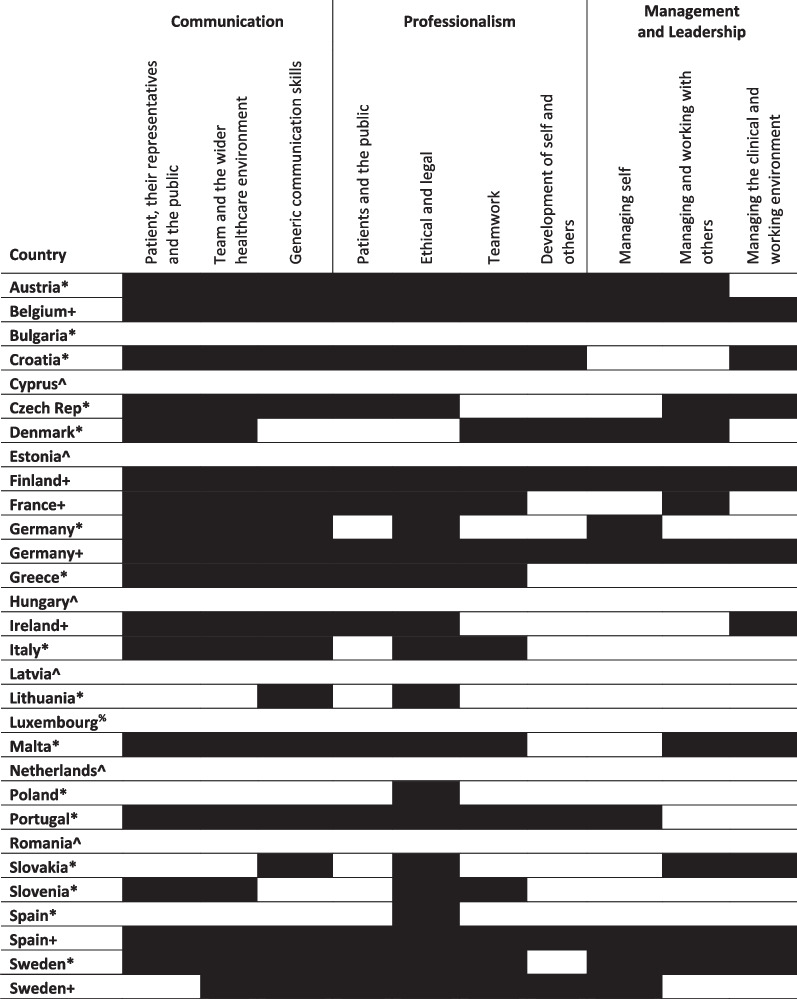
Mapping against communication, professionalism and management and leadership outcomes in preparing for practice

^+^Mapped to outcomes or curriculum from a national authority

*Mapped to outcomes or curriculum from a single dental school/university

^%^No dental school

^^^No information identified

Mapping the learning outcomes identified from across Europe to the GDC’s six overarching outcomes, shown in Table 1, revealed that there was evidence from most countries that their standards also covered the ‘demonstrate effective clinical decision-making’ and ‘apply an evidence-based approach to learning, practice, clinical judgement and decision-making and utilise critical thinking and problem solving’ standards set out in *Preparing for practice*. However, evidence that mapped to outcomes requiring graduating dentists to make ‘high quality long term care of patient the first concern’, ‘describe the principles of good research’ and ‘recognise the importance of life-long learning’ was found for less than half of EU member states. Only five countries had any information that covered graduates’ ability to ‘accurately assess their own capabilities and limitations’: Austria, Denmark, Finland, France and Sweden.

Below these overarching outcomes, the first subsection of *Preparing for practice* focuses on clinical skills relating to individual patient care and contains 13 broad criteria. As shown in Table 2, we identified seven countries where all these criteria were present as graduating outcomes: Croatia, Czech Republic, Germany, Greece, Ireland, Slovakia and Spain. Of these, data at national level was available for Germany, Ireland and Spain. Three other countries, Denmark, Malta and Slovenia, had only one missed outcome, as mapped at individual institution level. Outcomes relating to ‘Patient and public safety’, ‘Treatment of acute oral conditions’, ‘Management and treatment of periodontal disease’, and ‘Management of the developing and developed dentition’ were less likely to be included in outcome standards.

Table 3 shows our findings mapped against the outcomes expected in the communication, professionalism, and management and leadership domains of *Preparing for practice.* For some countries, mapped at institutional level only, very few outcomes were covered across these domains—this was the case for Bulgaria, Lithuania, and Poland. Conversely, there were some countries where all the outcomes across these three domains were found, namely Belgium, Finland, Germany, and Spain. All these countries had national level outcomes. Overall, of these three domains, communication and professionalism were well represented, but with less evidence for the outcomes focused on ‘teamwork’ and ‘development of self and others.’ The management and leadership domain was the least well evidenced, and only eight countries mapped to the ‘managing self’ outcome, or referenced a need for graduating dentists to be aware of their own limitations.

Finally, we found limited publicly  available information on quality assurance processes for dental education, although some information was provided by respondents to our questionnaire. In Ireland, dental education is accredited by the Dental Council. In most other responding countries (Belgium, Denmark, France, Portugal, Slovenia, Spain and Sweden) accreditation or quality assurance of dental education is not undertaken by a dental organisation, but by a national quality assurance agency for higher education.

## Discussion

By mapping curricula and learning outcomes for undergraduate dental training from EU member states against the learning outcomes set out for UK undergraduate dental education by the GDC in its *Preparing for Practice* document, our research demonstrates the challenges in comparing educational standards and expectations across national borders. We found that there was considerable variation between countries, where evidence of curricula or learning outcomes could be identified. This reflects the focus of the EU’s Directives on mutual recognition of professional qualifications on the duration of training rather than its content [15]. Determining the content of health professions education programmes remains the responsibility of national or institutional bodies.

Generally, we found there was better coverage across domains relating to clinical and communication skills. In some instances, data could be mapped to all or nearly all the domains and criteria of *Preparing for Practice.* However, we often could not establish if graduates had undertaken independent clinical work themselves or had been observing others work with patients. This is important as less exposure to direct treatment of patients before graduation may mean dentists entering the UK workforce without the levels of clinical experience expected by employers. Indeed, Davda et al. [16] found that some Internationally Qualified Dentists (IQDs), including from the EEA, practising in the UK recognised their different levels of experience in some clinical skills resulting from the content of their undergraduate training, negatively affected their ability to integrate into practice in the UK. Our systematic review of the literature also showed that the nature and extent of direct patient contact during training differed greatly across countries [17].

In other cases, we were unable to identify any publicly available information about dental curricula or learning outcomes, either at national or institutional levels. Where data were included at both institutional and national levels, we saw some differences in what could be mapped at each of these levels. Further research to more comprehensively examine the extent of these differences and the reasons for them would be useful. Variation between national and institutional levels illustrates the challenge facing decision-makers responsible for accrediting or recognising qualifications when that recognition is set at a national level, decreeing that all graduates from a given country are eligible to enter and practice in another national jurisdiction. National level recognition procedures may obscure differences in the educational provision offered by individual institutions. In most countries, dental education is not quality assured by a dental organisation.

While for some years there has been an educational agenda to harmonise dental education across Europe, it has been shown that there is little evidence of the extent to which harmonised curricula have actually been implemented. The dental education literature on this topic is dominated by proposals for curricula, unmatched by evaluative evidence of effective implementation [17]. ADEE’s *Profile and Competences for Graduating European Dentist,* originally published in 2005 [14], with updates in 2010 and 2017 [18, 19], is a key text in efforts towards cross-national educational harmonisation and we did find this document in use as the basis for national outcomes in Ireland and Finland, and references to its use at institutional level in Cyprus and Greece. Overall though, our findings show that there remains considerable variation between countries and institutions in the outcomes set for dental education in Europe.

Our findings illustrate the limitations of available data, and the need for further comparative work to achieve greater insights. An on-going project to improve the comparative data available about oral health professions education across Europe is seeking to collate information at programme level by collecting data directly from individual education providers [20]. That this work is necessary reflects the paucity of data currently available, as identified by our own research.

Getting recognition processes for out of country qualifications right is important for a number of reasons. First, to maintain patient safety by ensuring that only health professionals who are safe to practice effectively in a jurisdiction are able to do so. Variations in source country education have been identified as a factor IQDs reported confidence in their ability to undertake dental procedures when commencing work in the UK [16]. Furthermore, recognition processes need to ensure that countries can recruit the international workforce they need, and that health workers seeking to migrate are neither deterred nor penalised by overly prescriptive processes, as registration processes have been found to be a barrier to integration for healthcare workers migrating to the UK [21]. However, shifting recognition processes to the level of the institution also poses problems, in the form of increased bureaucratic burden and costs for bodies responsible for these processes, with those costs often passed on to individual practitioners through registration fees.

The impact of *Brexit* on the migration patterns of dentists, and other health professionals, to the UK is yet to be fully understood, and effects so far have been confounded by the concurrent impacts of the Covid-19 pandemic. However, the potential for post-Brexit accreditation and registration arrangements to impact on the dental workforce is clear. A 2019 survey of European-qualified dental professionals working in the UK found that Brexit was a significant factor for those who were considering leaving the UK, and identified concerns over the continuation of their rights to live and work there [22]. Continuation of the mutual recognition system for professional qualifications was reported as being the action most likely to dissuade dentists from leaving the UK [22].

While its longer term impacts on the healthcare workforce migration to the UK remain to be seen, it is certainly the case that exiting the EU has raised the issue of how to best manage the recognition of international qualifications. Beyond the national context of legislative changes brought about by Brexit, our analysis shows this issue is pertinent across Europe [15] and it is also relevant internationally. Developing processes that will allow reliable comparisons of curricula and learning outcomes, necessary to inform decisions about accreditation and potential additional training needs for IQDs, requires data about current education provision. However, our findings illustrate the limitations of available data for comparing curricula and learning outcomes across European countries, shortcomings also identified elsewhere [23].

### Limitations

This paper reports an attempt to map national level curricula and learning outcomes for dental education across Europe, using primarily publicly available information and also drawing on information provided by stakeholders. However, it is not an exhaustive mapping of all dental schools in each country, and where institution-level data are given this is intended as an example only. Our findings do not, therefore, necessarily reflect all aspects of how dental education is delivered in the countries included. In addition, there were some countries for whom data could not be identified, but this does not mean that those countries do not have national curricula or learning outcomes for dentistry. We were unable to ascertain quality assurance processes in every country but this does not mean they do not exist. Our mapping has used the *Preparing for Practice* framework [13], which reflects the UK context. It has its roots in the same harmonisation agenda as the ADEE *Profile and Competences for Graduating European Dentist* [14] and provides a structure upon which to base the comparison between countries.

## Conclusion

Against a backdrop of considerable change in the European cross-national policy landscape, and the residual uncertainty about how processes for the recognition of qualifications will operate in future, this paper provides a timely analysis of the extent to which basic dental training across Europe and the United Kingdom is demonstrably comparable. Offering insights into how effectively dental education can be compared at national level, as assumed in the model underpinning existing mutual recognition processes, our analysis aims to inform discussions about cross-national recognition of healthcare professionals’ qualifications, the regulation of health professions, and healthcare worker migration.

## Data Availability

The datasets used and/or analysed during the current study are available from the corresponding author on reasonable request.
